# A Clinical Early Evaluation of the Combined Use of Low‐Intensity Focused Ultrasound and Radiofrequency for Female Abdominal Contouring

**DOI:** 10.1111/jocd.70267

**Published:** 2025-07-17

**Authors:** Zongzhou Wu, Yun Wang, Wei Li, Wei Zhang, Lian Zhu

**Affiliations:** ^1^ Department of Cosmetology, Shanghai Skin Disease Hospital, School of Medicine Tongji University Shanghai China; ^2^ Department of Hematology Shanghai Putuo District Central Hospital Shanghai China; ^3^ Department of Radiation Oncology Shanghai East Hospital, School of Medicine, Tongji University Shanghai China

**Keywords:** aesthetic medicine, cosmetic dermatology, cosmetic surgery

## Abstract

**Background:**

The growing incidence of adiposity has driven the innovation of potent non‐invasive body contouring methodologies. Of these, low‐intensity focused ultrasound (LIFU) therapy is recognized for its precision in targeting and destroying stubborn adipose tissue. This study aims to assess the treatment effectiveness and safety of utilizing LIFU in conjunction with radiofrequency for female abdominal contouring.

**Methods:**

A prospective clinical trial was performed with a cohort of 20 women, aged 28–42 years, who had a body mass index ranging from 20.40 to 26.82 kg/m^2^ and exhibited abdominal skin laxity. We used LIFU and radiofrequency treatments, administered in six sessions over 6 weeks. Efficacy was assessed by measurements of abdominal circumferences using tape, subcutaneous adipose tissue thickness by ultrasound and computed tomography, dermal thickness using ultrasound, and abdominal appearance by standardized photography. The safety was evaluated using pain intensity assessment, nine hematological markers, and adverse events in abdominal appearance.

**Results:**

The treatment led to statistically significant reductions in supraumbilical, periumbilical, and subumbilical abdominal circumferences by 2.08 ± 3.29 cm (2.51%), 2.30 ± 3.23 cm (2.59%), and 3.03 ± 3.39 cm (3.29%), respectively. Similarly, significant decreases were observed in subcutaneous adipose tissue thickness at these sites by 6.46 ± 6.33 mm (27.37%), 6.03 ± 5.95 mm (23.28%), and 8.54 ± 6.94 mm (35.43%). On the other hand, dermal thickness demonstrated a mild increase of 9.17%, and none of the subjects experienced severe pain during the treatment sessions. Hematologic markers remained stable, and no adverse events were reported throughout the treatment period.

**Conclusions:**

This investigation established that implementing low‐intensity focused ultrasound alongside radiofrequency resulted in a secure and effective approach for abdominal contouring, contributing a novel strategy to medical practice.

## 1 Introduction

Adiposity often arises from a combination of factors, including aging, insufficient physical activity, imbalanced diet, and genetic predispositions. Obesity and overweight not only compromise physical aesthetics but also serve as significant risk factors for various chronic diseases, even amplifying the inherent mortality risk associated with these ailments [1, 2]. The abdominal region is often the primary site of adipose tissue accumulation. Even with attempts at weight loss through exercise and other means, the reduction of abdominal adipose tissue, a type of localized adiposity, remains particularly resistant to eradication and is commonly referred to as stubborn adipose tissue. With the advancement of medical technology and the escalating demand for aesthetic improvements, an increasing number of people are turning to body contouring technologies to improve their body shape, exhibiting significant benefits in the removal of localized adipose tissue.

Liposuction is a pioneer medical technique for body contouring, which surgically enables effective removal of subcutaneous adipose tissue. Nevertheless, it is an invasive approach potentially accompanied by the risks of adverse events, such as postoperative infection, scarring, prolonged recovery time, and deep vein thrombosis. Given these potential risks, a variety of non‐invasive body contouring technologies have been developed, including cryolipolysis [3, 4, 5], radiofrequency ablation [6], low‐level external laser therapy, injection lipolysis, high‐intensity focused electromagnetic technology [7], and focused ultrasound therapy [8, 9, 10]. Among these, focused ultrasound therapy has garnered significant attention in recent years. It focuses an ultrasonic beam precisely on the therapeutic region within the body for adipose tissue disruption, while sparing the surrounding normal tissue from damage. Moreover, it also stimulates the degeneration and contraction of collagen fibers and promotes the formation of new collagen, thereby achieving skin tightening. The technology is primarily categorized as high‐intensity focused ultrasound (HIFU) and low‐intensity focused ultrasound (LIFU), based on differences in intensity and frequency. Specifically, LIFU uses low‐intensity ultrasound to initiate a process of cyclic compression and expansion in the adipose tissue, producing an inertial cavitation effect [11]. This is the rapid expansion and abrupt collapse of bubbles under rapidly varying pressure, resulting in mechanical stress that effectively disrupts adipose cells. Additionally, the relative decrease in heat produced by LIFU enhances the comfort of subjects during the treatment session. UltraShape Power System (Syneron Medical Ltd., Yokneam, Israel) is currently the most prevalent LIFU device and has been approved by the Food and Drug Administration, Conformité Européenne, and the Israeli Ministry of Health. The device includes a hemispherical transducer and a main console that projects focused ultrasonic energy of a low frequency (200 ± 30 kHz) and low power density (1.75 W/cm^2^) to a predetermined depth, focusing the energy towards subcutaneous adipose tissue, while maintaining reduced energy at the skin surface. Pulsed ultrasound transmission used by the UltraShape Power allows for controlled temperature rise to achieve non‐thermal effects. While focusing solely on LIFU for abdominal lipolysis, there still persists the issue of abdominal laxity due to reduced muscular and fascial tension, affecting the contouring of the abdomen. Radiofrequency ablation can stimulate the growth of collagen and elastic fibers through deep heating, thereby achieving a skin‐tightening effect, which complements the role of focused ultrasound therapy in abdominal contouring. Previous research has indicated that the combined use of these two contouring technologies can enhance therapeutic outcomes and improve treatment efficiency [12, 13, 14, 15, 16].

We conducted a comprehensive evaluation of the application of LIFU in combination with radiofrequency in the field of female abdominal contouring. The treatment efficacy and safety of this approach were assessed by analyzing changes in abdominal circumferences using tape, subcutaneous adipose tissue thickness, and dermal thickness by ultrasound and computed tomography (CT) scans, standardized photography, pain intensity assessment, and hematological markers during the treatment process.

## 2 Materials and Methods

We conducted a prospective clinical trial involving a cohort of 20 female subjects. The enrollment criteria included: females aged between 28 to 42 years; body weight ranging from 50 to 75 kg (kg); and a body mass index (BMI) within the range of 20 to 30 kg per square meter (kg/m^2^); mild to moderate skin laxity in the abdominal area and a subcutaneous adipose tissue thickness of 2 to 3 cm as determined by CT scans. Exclusion criteria included individuals with a history of psychiatric and neurological disorders; those currently participating in other clinical trials; lactating or pregnant women; individuals with abdominal infections or scarring; presence of abdominal striae gravidarum; a history of abdominal hernia or tumorous conditions, a history of abdominal surgery and skin conditions in the abdominal area; individuals with chronic conditions such as heart disease, hypertension, pacemaker use, diabetes, lung diseases; and those with a history of smoking, alcohol abuse, and drug use. Subjects were withdrawn from subsequent treatment if abnormal liver function or lipid profiles occurred, particularly when values were threefold above the upper limit. Ethical approval was granted by the Ethics Committee of Shanghai Skin Disease Hospital. All subjects provided written consent, and treatments were performed at the Medical Aesthetics Department of the Affiliated Hospital of Dermatology of Tongji University.

The primary endpoint of the study was the assessment of the treatment efficacy of body contouring, including abdominal circumference measurements with a tape measure, subcutaneous abdominal adipose thickness measured by ultrasound, standardized photography, and CT scans. The secondary endpoint was dermal thickness by ultrasound and the safety evaluation, which included pain intensity assessment and testing of nine hematological markers.

**FIGURE 1:**
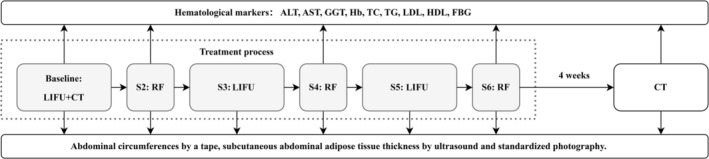
Flowchart of a treatment sequence alternating low‐intensity focused ultrasound (LIFU) and radiofrequency (RF) over six treatment sessions (S1–S6), followed by a CT scan after 4 weeks. Pain intensity assessment and hematological markers (upward arrows) as well as abdominal assessments (downward arrows) are performed at specified stages.

Each subject underwent six treatment sessions over a span of 6 consecutive weeks. The process flow of the treatments and evaluation is depicted in Figure 1. The treatment protocol was implemented as follows: The UltraShape system was administered for lipolysis, and the treatment areas were divided into four sections, each with 3–5 focal zones. Treatment is applied using the U‐Sculpt/VDF 660 W/cm^2^ transducer. With the subjects positioned supine, the treatment was executed for a duration of 45–60 min for each session. The iFine handpiece (EndyMed Medical Ltd., Caesarea, Israel) was employed to execute radiofrequency therapy for skin tightening. The preset power was set at 30 W, with a preheating target temperature of approximately 40°C. Each divided treatment area included eight 30‐s passes. No analgesics were administered during any stage of the treatment process. Subjects maintained their regular dietary and exercise habits during the treatment period.

Six methods were utilized for treatment evaluation. First of all, measurements of abdominal circumferences at supraumbilical, periumbilical, and subumbilical sites, as defined by three transverse finger‐widths above, middle, and below the navel circumference, were taken using a flexible tape. Subsequently, B‐mode ultrasound facilitated the estimation of abdominal subcutaneous adipose tissue thickness and dermal thickness by ultrasound. For the ultrasound, a portable ultrasound machine (Mindray DP‐10, Nanshan, Shenzhen, China) was employed, with measurements taken post‐application of a substantial gel layer. Software was utilized to calculate adipose tissue thickness between the dermis and muscular fascia at each of the abdominal sites above. Additionally, it facilitated the measurement of dermal thickness between the epidermis and the subcutaneous fat layer, specifically at the periumbilical region, with an average value calculated across three specified locations. The uCT 760 scanner (United Imaging, Shanghai, China) was utilized to perform CT scans at baseline and after 4 weeks of treatment to measure abdominal fat thickness. Additionally, standardized photographic documentation was executed with subjects in a standing position, and images were taken from three defined angles (0°, 45°, and 90°) in a clockwise direction, chronicling the aesthetic alterations in the abdominal area. Preliminary measurements obtained before the first treatment session were established as the baseline metrics for the study. The implementation of these evaluative procedures can be discerned from the process flow diagram as delineated in Figure 1. To evaluate the safety and compliance of the treatment, a questionnaire‐based Visual Analogue Scale (VAS) was employed to assess pain intensity, and a series of nine hematological markers was systematically examined, which comprise alanine aminotransferase (ALT), aspartate aminotransferase (AST), gamma‐glutamyl transferase (GGT), hemoglobin (Hb), total cholesterol (TC), triglycerides (TG), low‐density lipoprotein (LDL), high‐density lipoprotein (HDL), and fasting blood glucose (FBG).

Statistical analysis will be performed using the SPSS software (version 26.0). Metrics assessed at each visit will be statistically depicted as mean ± SD. Repeated measures analysis of variance will be adopted for analyzing within‐subject changes over time, with the level of significance threshold at *p* < 0.05. In the event of any missing data, we will employ a multiple imputation method to handle it.

## 3 Results

Between July and December 2022, we conducted a clinical trial involving 20 female subjects, aged 28 to 42 years (mean, 36.60 ± 3.61 years), with BMI values ranging from 20.40 to 26.82 kg/m^2^ (mean, 23.38 ± 1.99 kg/m^2^). All the subjects completed the prescribed sessions of treatment, which combined LIFU with radiofrequency.

### 3.1 Abdominal Circumferences by Tape

**FIGURE 2:**
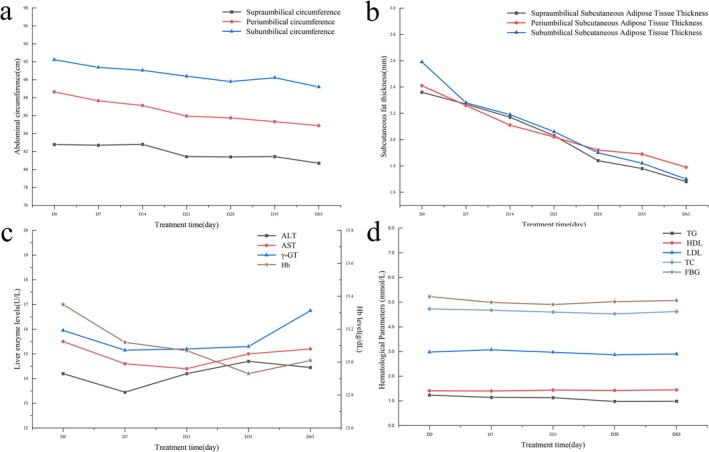
Assessment of therapeutic and biological outcomes from low‐intensity focused ultrasound and radiofrequency application for abdominal contouring. (a) Temporal variations in abdominal circumference by tape measurements, where the *y*‐axis signifies abdominal circumference (cm) and the *x*‐axis indicates treatment time with D0 as the baseline, and D14, D28, D35, and D63 corresponding to measurements on respective days and 4 weeks post‐treatment. (b) Measured alterations in subcutaneous adipose tissue thickness measured by B‐mode ultrasound, similarly depicted as in (a). (c) Changes in liver enzyme and hemoglobin levels, with the left *y*‐axis representing liver enzyme levels and the right *y*‐axis displaying Hb levels (g/dL), treatment time as in (a). (d) Fluctuations in blood lipid and glucose levels, where the left *y*‐axis displays total cholesterol, triglycerides, low‐density lipoprotein, and high‐density lipoprotein (mmol/L), and the right *y*‐axis signifies fasting blood glucose (mmol/L), following the same treatment time as in (a).

The baseline measurements for supraumbilical, periumbilical, and subumbilical abdominal circumferences, originally recorded as 82.78 ± 7.51 cm, 88.65 ± 6.95 cm, and 92.23 ± 6.48 cm, respectively, were observed to change to 80.7 ± 6.87 cm, 84.88 ± 6.46 cm, and 89.2 ± 6.09 cm after the treatment. As indicated by Table 1, the changes in supraumbilical, periumbilical, and subumbilical abdominal circumferences were 2.08 ± 3.29 cm (2.51%), 2.30 ± 3.23 cm (2.59%), and 3.03 ± 3.39 cm (3.29%). Concurrently, the temporal changes in abdominal circumferences, as demonstrated in Figure 2a, revealed a statistically significant reduction across supraumbilical, periumbilical, and subumbilical sites. Following a multivariate analysis employing one‐way and two‐way RM ANOVA, significant outcomes were observed. The one‐way RM ANOVA yielded *P* values of 0.042, 0.020, and 0.020, respectively, while the two‐way RM ANOVA demonstrated overall change as statistically significant (*p* = 0.009).

**TABLE 1:** Variations in abdominal circumferences and subcutaneous adipose tissue thickness post lipolysis combined with radiofrequency treatment.

Location	Analysis type	*F* ^a^	df^b^	*p* ^c^ ^s^	Change	PoC (%)
Abdominal circumferences by tape						
Supraumbilical	One‐way RM ANOVA	3.018	6, 14	0.042	2.08 ± 3.29 cm	2.51
Periumbilical		3.726	6, 14	0.020	2.30 ± 3.23 cm	2.59
Subumbilical		3.699	6, 14	0.020	3.03 ± 3.39 cm	3.29
Overall	Two‐way RM ANOVA	4.519	6, 14	0.009		
Subcutaneous adipose tissue thickness by B‐mode ultrasound						
Supraumbilical	One‐way RM ANOVA	3.861	6, 14	0.017	6.46 ± 6.33 mm	27.37
Periumbilical		3.398	6, 14	0.028	6.03 ± 5.95 mm	23.28
Subumbilical		6.272	6, 14	0.002	8.54 ± 6.94 mm	35.43
Overall	Two‐way RM ANOVA	5.151	6, 14	0.005		

Abbreviations: One‐way RM ANOVA, one‐way repeated measures analysis of variance; Two‐way RM ANOVA, two‐way repeated measures analysis of variance; PoC, proportion of change.

^a^ *F*‐statistic.

^b^ Df, degrees of freedom.

^c^ Probability value.

### 3.2 Subcutaneous Adipose Tissue Thickness and Dermal Thickness by Ultrasound

Initial ultrasound measurements revealed that the subcutaneous adipose tissue layer at the supraumbilical, periumbilical, and subumbilical sites had a thickness of 23.6 ± 6.8 mm, 25.9 ± 7 mm, and 24.1 ± 6.4 mm, respectively. Following the treatment, these measurements decreased to 16.8 ± 4.9 mm, 17.4 ± 5.5 mm, and 17.9 ± 5 mm. Corresponding reductions in thickness were found to be 6.46 ± 6.33 mm (27.37%), 6.03 ± 5.95 mm (23.28%), and 8.54 ± 6.94 mm (35.43%), respectively, detailed in Table 1.

**FIGURE 3:**
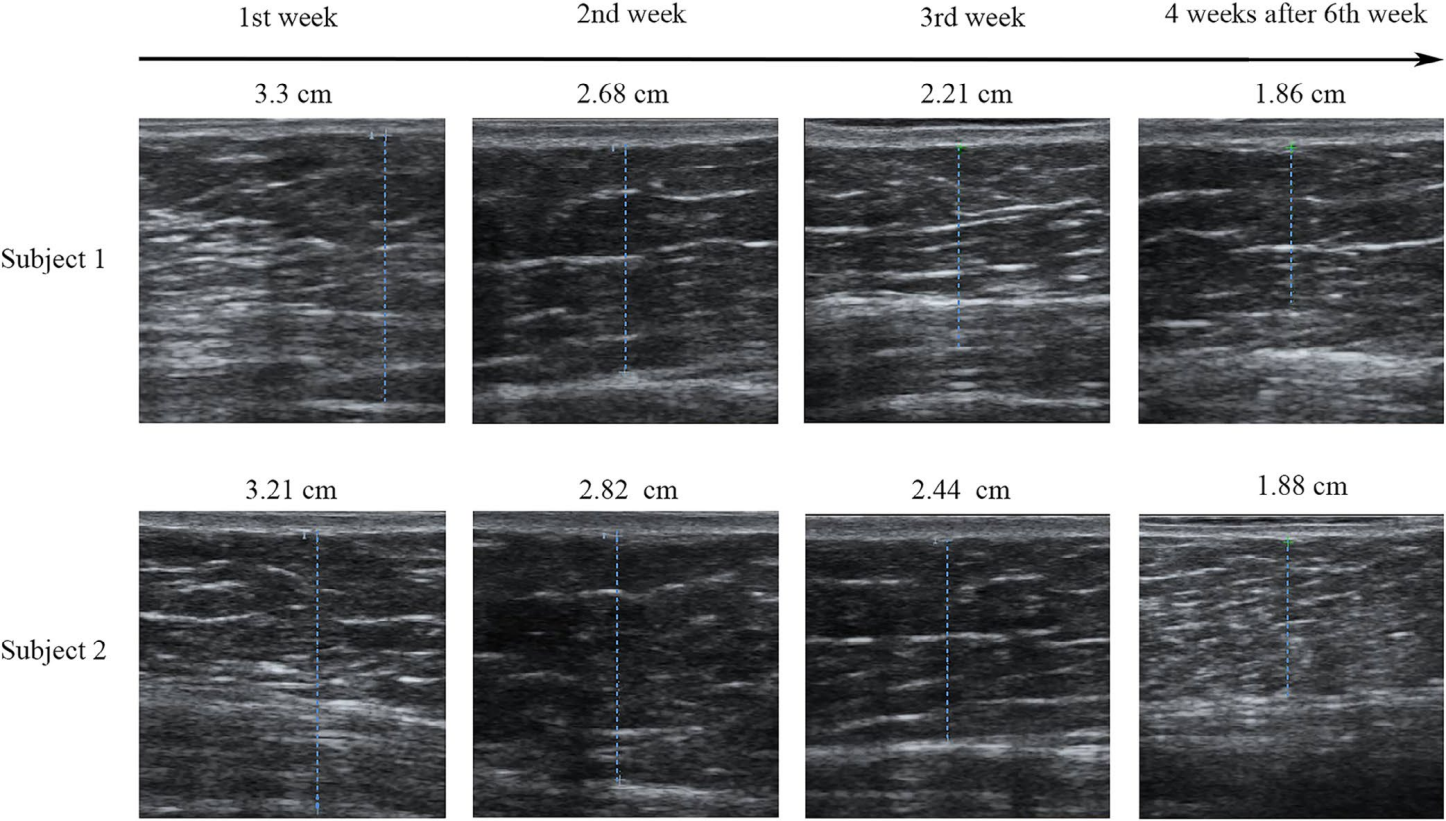
Ultrasonographic evaluation of subcutaneous adipose tissue thickness over time in two subjects. The figure shows ultrasonographic images of abdominal subcutaneous adipose tissue thickness at four time points (from left to right): 1st week, 2nd week, 3rd week, and 4 weeks after the 6th week. For Subject 1 (upper row), the thicknesses are 3.3 cm, 2.68 cm, 2.21 cm, and 1.86 cm, respectively. For Subject 2 (lower row), the thicknesses are 3.21 cm, 2.82 cm, 2.44 cm, and 1.88 cm, respectively.

Based on multivariate analysis, significant reductions in subcutaneous adipose tissue thickness were detected at the supraumbilical (*p* = 0.017), periumbilical (*p* = 0.028), and subumbilical (*p* = 0.002) sites via one‐way RM ANOVA. Additionally, a significant overall decrease was substantiated by the two‐way RM ANOVA (*p* = 0.005). Furthermore, ultrasound measurements of subcutaneous adipose tissue thickness also revealed a significant reduction over time, as presented in Figure 2b. The declining trend is further illustrated by Figure 3, which shows a sustained reduction in subcutaneous adipose tissue thickness for both subjects over the 4‐week period following the six treatment sessions. Subject 1 experienced a reduction from an initial measurement of 3.3 cm to 1.86 cm of subcutaneous adipose tissue thickness, while subject 2 showed a decrease from 3.21 cm to 1.88 cm.

According to the ultrasound measurements, the changes in dermal thickness before and after lipolysis combined with radiofrequency treatment are summarized in Table 2. This indicated an overall increase of 0.14 mm, corresponding to a mean 9.17% change (*p* < 0.05). Approximately 35% of all the subjects demonstrated an obvious increase in dermal thickness (> 20%), with a maximum change of 26.67%.

**TABLE 2:** Changes in dermal thickness measured by ultrasound.

Time	Thickness	Change	PoC^a^	*T* ^b^	*p* ^c^
1st week	1.47 ± 0.12 mm				—
4 weeks after the 6th week	1.60 ± 0.16 mm	0.14 ± 0.09 mm	9.17% ± 5.8%	6.95	0.0001

^a^ PoC, proportion of change.

^b^ *T*‐value.

^c^ Probability value.

### 3.3 Subcutaneous Adipose Tissue Thickness by CT

**FIGURE 4:**
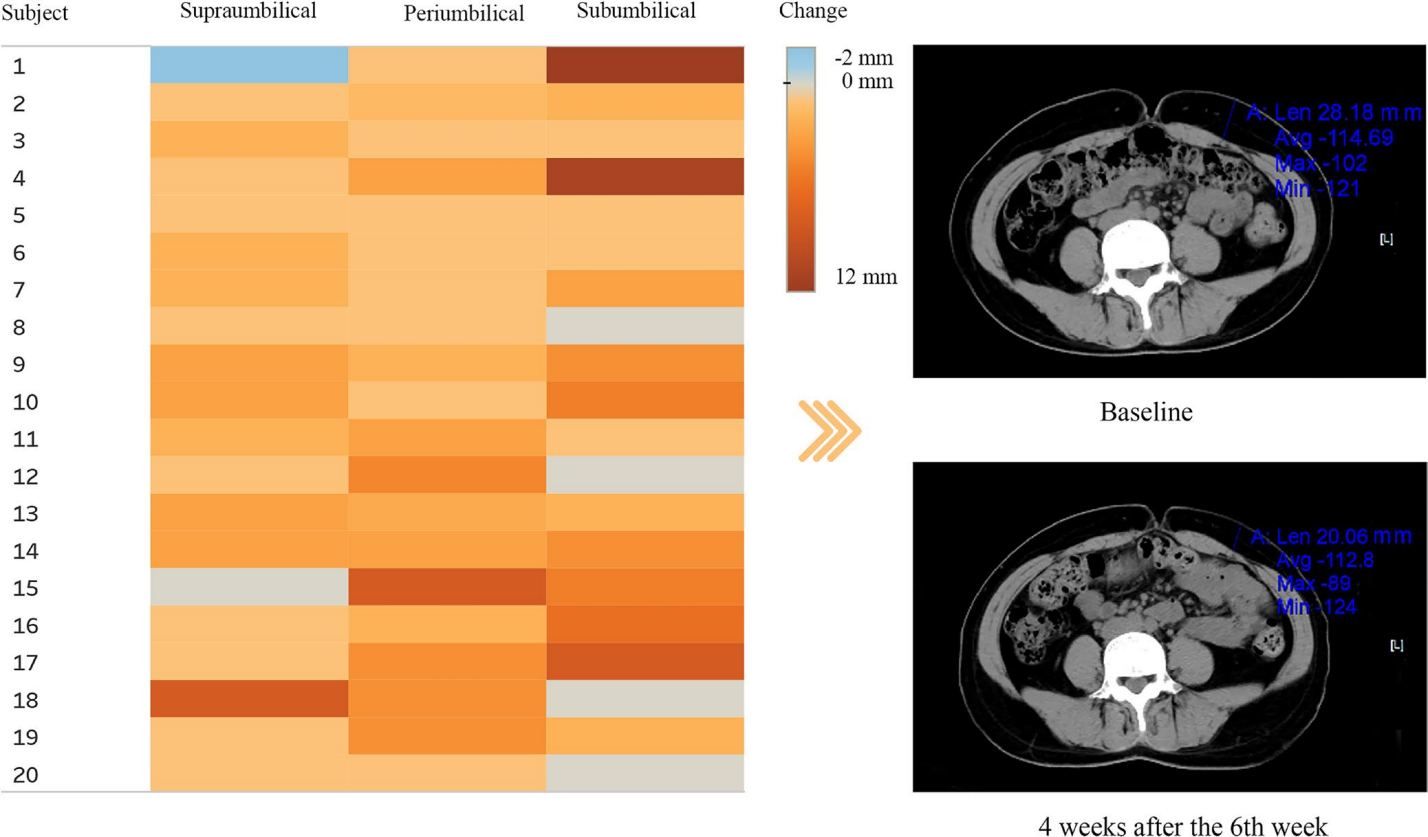
Heatmap and CT scans comparative display of subcutaneous adipose tissue thickness variation. The left visual depicts the subcutaneous adipose tissue thickness variability across 20 subjects (*y*‐axis) at three anatomical locations: Supraumbilical, umbilical, and subumbilical (*x*‐axis). The color intensity on the grid represents thickness change on a gradational scale from −2 cm (blue), 0 cm (gray), to 12 cm (deep red). The right top CT image shows the baseline subcutaneous adipose tissue thickness at 28.18 mm. The right bottom image, taken 4 weeks after the 6th week, illustrates a reduction in thickness to 20.06 mm. Both right images illustrate the variation in subcutaneous adipose tissue that corresponds to a single cell within the left heatmap, as observed in the CT scans.

The left heatmap signified that, while the majority of subjects exhibited significant thickness reduction (yellow to deep red gradient), a minor group either remained unchanged or exhibited an unexpected increase in adipose tissue thickness (blue to gray gradient) (Figure 4). The right individual subject images showed that the initial subcutaneous adipose tissue thickness of 28.18 mm at baseline diminished to 20.06 mm at a checkpoint 4 weeks subsequent to the six treatment sessions.

### 3.4 Standardized Photography

**FIGURE 5:**
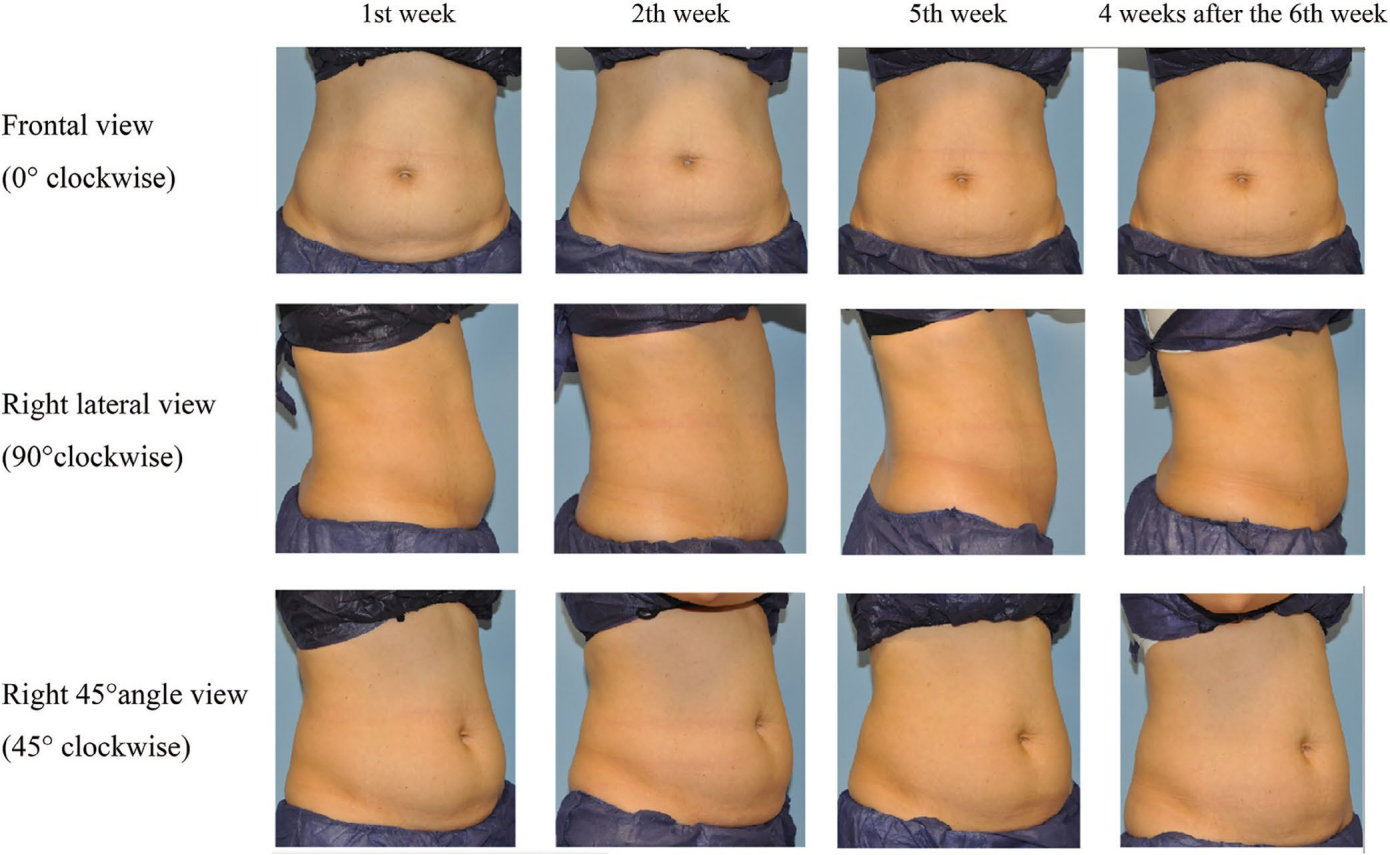
Temporal progression of abdominal appearance over 8 weeks. The figure presents three rows of images, demonstrating the temporal changes in abdominal appearance at the 1st, 2nd, 3rd, and 4 weeks after the 6th week. Views are represented in the order: the frontal (top row), the right lateral (middle row), and the right 45° angle (bottom row).

A standardized photographic method was employed to track alterations in abdominal appearance, revealing a consistent decrease in abdominal adipose tissue and an escalation in abdominal firmness. Moreover, none of the subjects exhibited adverse events of sensory abnormalities, hematoma, erythema, or swelling. Figure 5 provides a visual representation of the progressive changes, featuring standardized photographs taken from frontal view, right lateral view, and right 45° angle view at several time points (weeks 1, 2, 3, and 6) for two subjects. Compared to baseline, fewer visible skin folds and a smoother abdominal contour were noted at the 4‐week follow‐up after the sixth treatment session.

### 3.5 Pain Intensity Assessment

We evaluated the pain experienced by subjects during the combined LIFU and radiofrequency treatments for abdominal contouring. Pain levels were quantified using the VAS, which rates pain from 0 (no pain) to 10 (worst possible pain). Table 3 presents the pain intensity assessment results for LIFU and radiofrequency treatments across six sessions, with D0, D7, D14, D21, D28, and D35 representing treatment days 0, 7, 14, 21, 28, and 35, respectively. As shown in Table 3, the pain intensity remained consistently low across all sessions (VAS score ≤ 3). Notably, no cases of severe pain (VAS score ≥ 7) were reported among the subjects.

**TABLE 3:** Pain intensity scores of LIFU and radiofrequency.

Treatment	Time point	Mean ± SD	Minimum	Maximum
LIFU	D0	0.70 ± 0.71	0	2
	D14	0.65 ± 0.91	0	3
	D28	0.45 ± 0.67	0	2
	Overall	0.60 ± 0.78	0	3
Radiofrequency	D7	0.95 ± 1.02	0	3
	D21	0.85 ± 0.73	0	2
	D35	0.50 ± 0.59	0	2
	Overall	0.77 ± 0.82	0	3

### 3.6 Assessment of Hematologic Markers

According to the measured hematological markers, it indicated a maintained stability in the levels of liver enzymes, Hb, lipids, and glucose across the treatment period, as can be discerned from Figure 2c,d. Our multivariate analysis demonstrated a statistical insignificance in fluctuations of ALT, AST, and GGT with respective *P*‐values of 0.643, 0.569, and 0.114. Similarly, Hb, TC, TG, LDL, and HDL levels displayed *P*‐values of 0.066, 0.386, 0.301, 0.634, and 0.191. Notably, it was observed that FBG levels exhibited a statistically significant *P*‐value of 0.024. However, the minor decline from 5.23 mmol/L to 5.06 mmol/L did not seem to hold any substantial clinical implications.

## 4 Discussion

This study aims to assess the treatment effectiveness and safety of the clinical application of LIFU combined with radiofrequency for female abdominal sculpting. Studies on the utilization of focused ultrasound and radiofrequency for abdominal contouring treatments are comparatively scarce. Shek et al. incorporated 20 Asian subjects into their study, where low‐frequency non‐thermal focused ultrasound in conjunction with radiofrequency was employed for abdominal contouring treatments thrice a week for two weeks [17]. Significant reduction in average abdominal circumference was observed in 17 subjects who completed the full course of treatment (*p* = 0.023). Hugul et al. retrospectively analyzed 64 subjects who underwent low‐frequency ultrasound with single‐stage dual‐loop radiofrequency treatment for their thighs, arms, and abdomen [18]. All subjects underwent four treatment sessions, with significant changes recorded in the circumferences of the treated areas from the initial treatment to a week post‐treatment. A prospective, non‐randomized, open‐label study conducted by Urdiales‐Ga'lvez et al. involved the enrollment of 15 subjects who underwent four treatment sessions of non‐focused pulsed ultrasound combined with monopolar radiofrequency therapy, focusing on abdominal and gluteal regions [13]. The outcome of this study demonstrated a reduction in the Hodges‐Lehmann median differences for the areas below the navel, above the navel, right buttock, and left buttock.

Furthermore, post‐treatment adipose tissue volume decreased from an initial 32.9% to 31.2% (*p* = 0.0006), suggesting the therapy's efficacy in reducing adipose tissue thickness and its high safety and subject satisfaction rates. Distinct from these studies, our present study employed a more advanced multipolar radiofrequency ablation. This technology, unlike preceding unipolar and bipolar radiofrequency approaches, allows for more precise control over the treatment depth, uniform heat distribution, and enhances safety and therapeutic efficacy. Our study utilized a phase‐controlled multi‐source radiofrequency system, which is composed of an electrode array driven by six independent phase‐controlled radiofrequency generators. This system is capable of transmitting energy to a depth of 11 cm within the papillary dermis, reticular dermis, and the superficial fascial layer for localized heating, consequently stimulating the generation of new collagen and elastic fibers [19]. Moreover, compared to earlier studies, our study offers a more comprehensive evaluation of treatment efficacy and safety, including quantitative measurements of abdominal circumferences, multimodal imaging for displaying subcutaneous adipose tissue thickness and dermal thickness, standardized photos for recording the changes in abdominal appearance, pain intensity assessment, and the multiple hematological indices for safety assessment.

Our investigation demonstrated that the RM ANOVA of the abdominal circumferences measured by the cape at three sites (supraumbilical, periumbilical, and subumbilical) from the baseline to 4 weeks after the sixth treatment session indicated significant decreasing trends, with *P*‐values of 0.042, 0.020, and 0.020, respectively. Likewise, similar trends were observed in the thickness of the subcutaneous adipose tissue as measured by ultrasound, with the *P*‐values for the supraumbilical (*p* = 0.017), periumbilical (*p* = 0.028), and subumbilical (*p* = 0.002) sites. CT scan results further substantiated these observations. Figure 4 illustrates the variations in the thickness of subcutaneous adipose tissue across various sites for each subject by CT scans, as visualized using a heatmap. A dominance of areas varying from yellow to red, juxtaposed with only sporadic blue areas, suggests a significant reduction in abdominal adipose tissue thickness in the majority of subjects. Collectively, the evidence suggests that the combination of LIFU and radiofrequency is an effective approach for abdominal contouring, capable of reducing abdominal adipose tissue. Based on the tape and ultrasound measurements, a more pronounced change was observed at the subumbilical site compared to the other two sites. A possible explanation for this discrepancy could be the influence of gravity during tape and ultrasound measurements when these subjects were standing. Accounting for the influence of the factor in question, we have selected three different locations: supraumbilical, periumbilical, and subumbilical for our measurements. This permits a more reliable assessment of the variations in abdominal adipose tissue. Additionally, the supine position during CT scans may lead to a decrease in subcutaneous adipose tissue thickness in the abdominal region due to gravitational compression. As a result, CT measurement typically yields smaller values of subcutaneous adipose tissue thickness compared to those acquired through ultrasound methods, but a clear trend of reduction was nonetheless observed in our study.

The effectiveness of focused ultrasound for abdominal contouring is influenced to a considerable extent by factors such as baseline abdominal adipose tissue thickness, body frame, and the number and duration of treatments. Previous studies have reported significant variance in the reduction of abdominal adipose following LIFU treatment. Teitelbaum et al. conducted a multicenter clinical study, evaluating 164 subjects within 12 weeks after a single treatment using the same type of device [20]. The result indicated a decrease in abdominal circumference by 2.3 ± 0.32 cm. However, in a study by Shek et al., involving 53 subjects undergoing up to 3 times of body contouring treatments within a month, revealed an abdominal circumference reduction of 1.1056 ± 1.83863 cm and subcutaneous adipose tissue thickness reduction of 0.2769 ± 0.80775 cm, attributing the less substantial effect in Southeast Asians to smaller body frames [21]. The majority of subjects selected in our study are within the normal weight (BMI = 18.5–24.9 kg/m^2^) and a subcutaneous adipose tissue thickness of 2 to 3 cm, deemed more appropriate for localized fat reduction compared to overweight (BMI = 25–29.9 kg/m^2^) and obese (BMI > 30 kg/m^2^) individuals, leading to moderate baselines for abdominal circumferences and subcutaneous adipose tissue thickness. Similar to Southeast Asians, the smaller body frames of Chinese subjects also resulted in minimal changes in subcutaneous adipose tissue thickness in our study.

LIFU combined with radiofrequency treatment resulted in an average increase in dermal thickness of 0.14 mm (9.17%, *p* < 0.05). This effect is attributed to the transmission of radiofrequency energy into the dermis, where continuous heating induces fibroblast activation, thereby promoting the synthesis of new collagen and elastin fibers. These processes contribute to dermal thickening and enhanced skin elasticity. Correspondingly, the standardized photographs in Figure 3 illustrate a progressive reduction in adiposity alongside a visible enhancement in dermal firmness, highlighting the dual benefits of this treatment. Furthermore, the substantial variability in individual responses, with maximum increases in dermal thickness reaching 26.67%, suggests the potential influence of factors such as baseline skin condition, metabolic activity, and sensitivity to thermal energy on treatment outcomes.

In the design and execution of a clinical trial for a novel therapeutic approach, safety assessment is fundamental to evaluate feasibility. The results from Table 3 demonstrate that pain scores were consistently low, indicating that both LIFU and radiofrequency treatments are generally well‐tolerated with discomfort remaining at manageable levels. Our study involved not only monitoring potential skin reactions in subjects, such as pain, edema, bruising, erythema, and abnormal sensations, but also the inspection of certain hematological markers to assess physiological changes during therapy. The results showed steady levels of liver enzymes, hemoglobin, blood lipids, and glucose throughout the treatment period, substantiating the safety of this approach. Kiedrowicz et al. executed a study incorporating a cohort of 60 female subjects, using a focused ultrasound device operating at a frequency of 1 MHz and intensity of 1.4 W/cm^2^. Subjects were subdivided into three groups: ultrasound, radiofrequency, and the combined ultrasound‐radiofrequency treatment, in which the evaluation of various hematological markers was performed [16]. After 10 treatments and 6 months post‐treatment, each subgroup's peripheral blood leukocyte counts and fasting venous plasma glucose, insulin, C‐reactive protein, TC, low‐density lipoprotein cholesterol, high‐density lipoprotein cholesterol, and TG levels, along with levels of free fatty acids, were assessed. The results showed no direct impact on these lipid profiles, insulin resistance markers, inflammation markers, or risk factors for hypertension and cardiovascular disease, whether in the short or long term, aligning with our own hematological findings. However, their study employed more treatment sessions and a longer monitoring duration, further affirming the high safety of combined ultrasound‐radiofrequency treatment.

Nonetheless, our study is subject to some limitations. Firstly, the dietary habits of our subjects could potentially have introduced some level of interference with the results. Although we have made every effort to control potential confounders, their impact on our findings cannot be entirely ruled out. Additionally, the limited sample size of our study, though it demonstrated some statistical significance, might have restricted our capacity to reflect the true properties of the data. In our future research, we plan to extend this line of research by gathering a larger cohort, as well as incorporating a diet assessment questionnaire to capture dietary patterns. This inclusion of caloric intake as a determinant will allow for a more accurate evaluation of changes in abdominal adipose tissue.

## 5 Conclusions

Our study was designed to explore the clinical results of applying LIFU along with radiofrequency for abdominal contouring. The methods we used for evaluation involved measurements of abdominal circumferences using a tape measure, subcutaneous adipose thickness assessment by B‐mode ultrasound and CT scans, dermal thickness by B‐mode ultrasound, and the taking of standardized photographs. Additionally, we monitored pain intensity, certain hematological indicators, and adverse events in abdominal appearance. Our findings confirm the effectiveness and safety of this approach, providing a new option for abdominal contouring in medical practice.

## Author Contributions

Lian Zhu and Wei Zhang conceived the idea and conceptualized the study. Zongzhou Wu and Wei Li collected the clinical data. Yun Wang analyzed the data. Zongzhou Wu drafted the manuscript, and Lian Zhu revised the manuscript. All authors read and approved the final draft.

## Ethics Statement

This study was conducted in strict adherence to the ethical principles outlined in the Declaration of Helsinki and its later amendments. Ethical approval for the research was granted by the Institutional Review Board of Shanghai Skin Disease Hospital (Approval No. 2021–25 (Sci)) on August 30, 2021. Informed consent was duly obtained from all individual participants included in this study.

## Conflicts of Interest

The authors declare no conflicts of interest.

## Data Availability

The data that support the findings of this study are available on request from the corresponding author. The data are not publicly available due to privacy or ethical restrictions.

## References

[1] E. E. Calle , C. Rodriguez , K. Walker‐Thurmond , and M. J. Thun , “Overweight, Obesity, and Mortality From Cancer in a Prospectively Studied Cohort of U.S. Adults,” New England Journal of Medicine 348, no. 17 (2003): 1625–1638, 10.1056/NEJMoa021423. 12711737

[2] S. A. C. McDowell , S. Milette , S. Doré , et al., “Obesity Alters Monocyte Developmental Trajectories to Enhance Metastasis,” Journal of Experimental Medicine 220, no. 8 (2023): 20220509, 10.1084/jem.20220509. PMC10182775 37166450

[3] I. C. Hwang , K. K. Kim , and K. R. Lee , “Cryolipolysis‐Induced Abdominal Fat Change: Split‐Body Trials,” PLoS One 15, no. 12 (2020): e0242782, 10.1371/journal.pone.0242782. 33373395 PMC7771684

[4] E. Coiante , R. Pensato , I. Hadji , et al., “Assessment of the Efficacy of Cryolipolysis on Abdominal Fat Deposits: A Prospective Study,” Aesthetic Plastic Surgery 47 (2023): 2679–2686, 10.1007/s00266-023-03369-0. 37138191

[5] A. K. Rzepecki , A. S. Farberg , P. W. Hashim , and G. Goldenberg , “Update on Noninvasive Body Contouring Techniques,” Cutis 101, no. 4 (2018): 285–288. 29763486

[6] R. A. Weiss , “Noninvasive Radio Frequency for Skin Tightening and Body Contouring,” Seminars in Cutaneous Medicine and Surgery 32, no. 1 (2013): 9–17. 24049924

[7] J. B. Samuels , B. Katz , and R. A. Weiss , “Radiofrequency Heating and High‐Intensity Focused Electromagnetic Treatment Delivered Simultaneously: The First Sham‐Controlled Randomized Trial,” Plastic and Reconstructive Surgery 149, no. 5 (2022): 893e–900e, 10.1097/prs.0000000000009030. PMC9028295 35259147

[8] M. L. Jewell , N. J. Solish , and C. S. Desilets , “Noninvasive Body Sculpting Technologies With an Emphasis on High‐Intensity Focused Ultrasound,” Aesthetic Plastic Surgery 35, no. 5 (2011): 901–912, 10.1007/s00266-011-9700-5. 21461627

[9] D. Mazzoni , M. J. Lin , D. P. Dubin , and H. Khorasani , “Review of Non‐Invasive Body Contouring Devices for Fat Reduction, Skin Tightening and Muscle Definition,” Australasian Journal of Dermatology 60, no. 4 (2019): 278–283, 10.1111/ajd.13090. 31168833

[10] N. Y. Lee and D. M. Robinson , “Noninvasive Body Contouring,” Seminars in Cutaneous Medicine and Surgery 36, no. 4 (2017): 170–178, 10.12788/j.sder.2017.043. 29224034

[11] R. S. Mulholland , M. D. Paul , and C. Chalfoun , “Noninvasive Body Contouring With Radiofrequency, Ultrasound, Cryolipolysis, and Low‐Level Laser Therapy,” Clinics in Plastic Surgery 38, no. 3 (2011): 503–520, 10.1016/j.cps.2011.05.002. 21824546

[12] V. C. Canela , C. N. Crivelaro , L. Z. Ferla , et al., “Synergistic Effects of Combined Therapy: Nonfocused Ultrasound Plus Aussie Current for Noninvasive Body Contouring,” Clinical, Cosmetic and Investigational Dermatology 11 (2018): 203–212, 10.2147/ccid.S157782. 29731654 PMC5927144

[13] F. Urdiales‐Gálvez , S. Martín‐Sánchez , M. Maíz‐Jiménez , and E. Viruel‐Ortega , “Body Contouring Using a Combination of Pulsed Ultrasound and Unipolar Radio Frequency: A Prospective Pilot Study,” Aesthetic Plastic Surgery 46, no. 5 (2022): 2438–2449, 10.1007/s00266-022-02919-2. 35648192 PMC9159040

[14] A. Michon , “A Prospective Study Determining Patient Satisfaction With Combined Cryolipolysis and Shockwave Therapy Treatment for Noninvasive Body Contouring,” Aesthetic Plastic Surgery 45, no. 5 (2021): 2317–2325, 10.1007/s00266-021-02139-0. 33515083

[15] S. J. Theodorou , R. J. Paresi , and C. T. Chia , “Radiofrequency‐Assisted Liposuction Device for Body Contouring: 97 Patients Under Local Anesthesia,” Aesthetic Plastic Surgery 36, no. 4 (2012): 767–779, 10.1007/s00266-011-9846-1. 22466060

[16] M. Kiedrowicz , E. Duchnik , J. Wesołowska , et al., “Early and Long‐Term Effects of Abdominal Fat Reduction Using Ultrasound and Radiofrequency Treatments,” Nutrients 14, no. 17 (2022): 3498, 10.3390/nu14173498. 36079758 PMC9459719

[17] S. Y. Shek , C. K. Yeung , J. C. Chan , and H. H. Chan , “The Efficacy of a Combination Non‐Thermal Focused Ultrasound and Radiofrequency Device for Noninvasive Body Contouring in Asians,” Lasers in Surgery and Medicine 48, no. 2 (2016): 203–207, 10.1002/lsm.22406. 26352171

[18] H. Hugul , M. C. Oba , and Z. Kutlubay , “Efficacy of Focused Radiofrequency With Ultrasound in Body Contouring: A Study of 64 Patients,” Journal of Cosmetic Dermatology 20, no. 8 (2021): 2507–2511, 10.1111/jocd.13896. 33340220

[19] L. Gao , H. Kang , Y. Li , et al., “Clinical Efficacy and Safety of 3DEEP Multisource Radiofrequency Therapy Combined With Fractional Skin Resurfacing for Periocular Skin Aging,” Journal of Clinical and Aesthetic Dermatology 13, no. 3 (2020): 41–44. PMC7159313 32308797

[20] S. A. Teitelbaum , J. L. Burns , J. Kubota , et al., “Noninvasive Body Contouring by Focused Ultrasound: Safety and Efficacy of the Contour I Device in a Multicenter, Controlled, Clinical Study,” Plastic and Reconstructive Surgery 120, no. 3 (2007): 779–789, 10.1097/01.prs.0000270840.98133.c8. 17700131

[21] S. Shek , C. Yu , C. K. Yeung , T. Kono , and H. H. Chan , “The Use of Focused Ultrasound for Non‐Invasive Body Contouring in Asians,” Lasers in Surgery and Medicine 41, no. 10 (2009): 751–759, 10.1002/lsm.20875. 20014261

